# Localization of decorin gene expression in normal human breast tissue and in benign and malignant tumors of the human breast

**DOI:** 10.1007/s00418-012-1026-0

**Published:** 2012-09-25

**Authors:** Pia Boström, Annele Sainio, Tanja Kakko, Mikko Savontaus, Mirva Söderström, Hannu Järveläinen

**Affiliations:** 1 Department of Pathology, Turku University Hospital, University of Turku, Kiinamyllynkatu 4-8, 20520 Turku, Finland; 2 Department of Medical Biochemistry and Genetics, University of Turku, Kiinamyllynkatu 10, 20520 Turku, Finland; 3 Department of Medicine, Turku University Hospital, Kiinamyllynkatu 4-8, 20520 Turku, Finland

**Keywords:** Breast cancer, Extracellular matrix, Decorin, In situ hybridization, Cell behavior

## Abstract

The small extracellular matrix proteoglycan decorin which possesses a potent antitumor activity has been shown to be present in various amounts in the stroma of several tumors including those of the breast. Regarding decorin in breast malignancies the published data are conflicting, i.e., whether breast cancer cells express it or not. Here, we first compared decorin gene expression levels between healthy human breast tissue and selected types of human breast cancer using GeneSapiens databank. Next, we localized decorin mRNA in tissue specimen of normal human breast, intraductal breast papillomas and various histologic types of human breast cancer using in situ hybridization (ISH) with digoxigenin-labeled RNA probes for decorin. We also examined the effect of decorin transduction on the behavior of cultured human breast cancer MCF7 cells. Analysis of GeneSapiens databank revealed that in various human breast cancers decorin expression is significant. However, ISH results clearly demonstrated that human breast cancer cells independently of the type of the cancer do not express decorin mRNA. This was also true for papilloma-forming cells of the human breast. Indeed, decorin gene expression in healthy human breast tissue as well as in benign and malignant tumors of human breast was shown to take place solely in cells of the original stroma. Decorin transduction using decorin adenoviral vector in decorin-negative MCF7 cells resulted in a significant decrease in the proliferation of these cells and changed cell cohesion. Decorin-transduced MCF7 cells also exhibited increased apoptosis. In conclusion, our study shows that in human breast tissue only cells of the original stroma are capable of decorin gene expression. Our study also shows that transduction of decorin in decorin-negative human breast cancer cells markedly modulates the growth pattern of these cells.

## Introduction

In recent years, growing interest has been focused on the role and therapeutic potential of extracellular matrix (ECM) macromolecules in various diseases including the evolution and progression of cancer (Hielscher et al. 2012; Järveläinen et al. 2009; Lu et al. 2012; Marastoni et al. 2008). This is because today we understand that ECM macromolecules form not only an inert, space-filling microenvironment around the cells but they also interact with cells and generate signals that regulate the behavior of the cells (Hynes 2009; Rozario and DeSimone 2010) and control angiogenesis (Hielscher et al. 2012). Indeed, individual ECM macromolecules display important functional roles in the control of key cellular events of physiologic and pathological processes, namely adhesion, migration, proliferation, differentiation, and survival (Daley et al. 2008; Järveläinen et al. 2009; Marastoni et al. 2008; Rozario and DeSimone 2010).

An ECM molecule that has been shown to be involved in the regulation of all the aforementioned cellular events, and thereby markedly contributes to health and disease, is decorin, a small leucine-rich extracellular matrix proteoglycan (Ferdous et al. 2010; Iozzo and Schaefer 2010; Seidler and Dreier 2008). The evidence suggests that decorin represents a potent antitumor molecule (Iozzo and Sanderson 2011; Theocharis et al. 2010). For example, early studies with decorin-deficient mice have indicated that although the lack of decorin does not lead to the development of spontaneous tumors (Danielson et al. 1997), it is permissive for tumorigenesis (Iozzo et al. 1999). In concordance with this, we have recently demonstrated that there is a striking difference in the expression of decorin between malignant and benign vascular tumors in human, i.e., within Kaposi’s sarcoma, and angiosarcoma, decorin expression is completely lacking; while within hemangiomas, decorin is expressed in abundant amounts (Salomäki et al. 2008). Decorin has also been shown to inhibit tumor growth by antagonizing tumor angiogenesis (Neill et al. 2012). Furthermore, ectopic expression of decorin has been shown to cause generalized growth suppression in neoplastic cells of various histologic origin (Santra et al. 1997). As can be expected, several other studies supporting the antitumor and antimetastatic activity for decorin have been published (Biaoxue et al. 2011; Goldoni and Iozzo 2008; Hu et al. 2009; Reed et al. 2005; Shintani et al. 2008; Troup et al. 2003), and low levels of decorin have been found to be associated with a shorter progression time and poorer survival in lymph node-negative invasive human breast carcinomas (Araki et al. 2009; Troup et al. 2003). As such, a lot of interest has been paid to the potential use of decorin as an anticancer agent in the future (Neill et al. 2012; Pucci-Minafra et al. 2008; Theocharis et al. 2010).

Breast cancer is the leading cancer malignancy among women aged 20–59 years (WHO 2009). It comprises a collection of diseases that have different histopathology, genetic, and genomic variations, and prognostic outcomes (Geyer et al. 2009). In both benign and malignant breast tumors, alterations of stromal structure and composition are well-recognized phenomena (Alowami et al. 2003; Brown et al. 1999; Lu et al. 2012). These alterations in turn are likely to play an important role in the growth and invasion of breast lesions (Brown et al. 1999; Lu et al. 2012). Regarding decorin, its expression has been shown to be increased in the peritumoral stroma of the malignant lesions but decreased within the breast tumor tissue (Brown et al. 1999; Leygue et al. 2000). However, results on decorin expression in human breast cancer have been somewhat conflicting, i.e., whether breast cancer cells express this small proteoglycan or not (Cawthorn et al. 2012; Gu et al. 2010; Leygue et al. 2000; Skandalis et al. 2011). Thus, in the present study, we aimed to localize decorin mRNA in individual cells within normal and malignant human breast tissues using in situ hybridization (ISH) with digoxigenin (DIG)-labeled RNA probes for decorin. In addition, by utilizing cultures of MCF7 human breast adenocarcinoma cells and a decorin producing adenoviral vector, we also examined whether targeted decorin delivery can modulate the behavior of these cells.

## Materials and methods

### Patients and tumors

Ethical approval for the use of the clinical material of this study was given by Turku University Hospital Ethics committee (no 241/2005) and the Finnish National Authority for Medicolegal Affairs (no 4424/32/300/02). Well-characterized human breast cancer material consisted of tumor samples collected from 69 female breast cancer patients (mean age at surgery 65.4 years, range 40–94 years) who were operated and treated at Turku University Hospital during the years 2004–2007 (Table 1). All patients had over 10 mm invasive breast tumor and were treated with a radical mastectomy. Tissue samples from the invasive border of the tumor were excised within 30 min after the surgical removal of the breast. Normal human female breast tissue (three samples) and intraductal papillomas (three samples) were obtained through reduction mammoplasty of the healthy side. The specimen were fixed in 10 % phosphate buffered formaldehyde and embedded in paraffin. Four μm serial sections were cut and stained with hematoxylin and eosin. The slides were reviewed to confirm the diagnosis of the breast cancer, and the histologic typing and grading of the specimen were performed according to the World Health Organization (WHO) classification (Ellis et al. 2003). Of the 69 patients, 25 cases were grade III tumors, 36 cases were grade II tumors, and 8 cases were grade I tumors. Histologically, the majority were invasive ductal carcinomas (71 %). Lymph node metastases were found in 33 cases. Estrogen receptors (ER) were determined positive in 56 cases and progesterone receptors (PR) were positive in 53 cases. Ki-67 status was intermediate (16–30 %) or high (>30 %) in 46 cases. Among the 69 cases of invasive breast cancer specimen studied, Her2 chromogenic in situ hybridization (CISH) positivity was found in 11 patients.

**Table 1:** Patients and tumor characteristics

Variable	Number of patients (%)
Number of the patients	69 (aged 40–94, mean 65.4)
Grade	
I	8 (11.6 %)
II	36 (52.2 %)
III	25 (36.2 %)
Axillary nodal status	
N0	33 (47.8 %)
>N1	33 (47.8 %)
Unknown	3 (4.3 %)
Tumor size	
≤2 cm	23 (33.3 %)
>2 cm	46 (66.7 %)
Estrogen receptor status (ER)^a^	
Positive	56 (81.2 %)
Negative	13 (18.8 %)
Progesterone receptor status (PR)^a^	
Positive	53 (76.8 %)
Negative	16 (23.2 %)
Ki-67 status^b^	
Low ≤15 %	23 (33.3 %)
Intermediate 16–30 %	26 (37.7 %)
High >30 %	20 (29 %)
Histologic type	
Ductal	49 (71.0 %)
Lobular	11 (15.9 %)
Subtypes	9 (13.0 %)
Her2^c^	
IHC positive (2+ and 3+)	27 (39.1 %)
IHC negative (0 and 1+)	42 (60.9 %)
CISH positive	11 (15.9 %)

^a^Cut off point used for ER and PR immunohistochemistry is 10 % of positively stained tumor nuclei.

^b^Proliferation index according to St Gallen Consesus (Goldhirsch et al. 2009)

^c^Scoring of HER2 immunohistochemistry: *Score 0* no staining is observed or cell membrane staining is observed in less than 10 % of tumor cells. *Score 1+* a faint perceptible membrane staining can be detected in more than 10 % of the tumor cells or cells are only stained in part of their membrane. *Score 2+* a weak-to-moderate complete membrane staining is observed in more than 10 % of the tumor cells. *Score 3+* a strong complete membrane staining is observed in more than 10 % of the tumor cells

### GeneSapiens database

The GeneSapiens database was used to analyze previous published results of the gene expression levels of decorin in healthy and malignant human breast tissues (Kilpinen et al. 2008). This database (http://www.genesapiens.org/) covers the relative gene expression patterns for 17,330 genes across all the 9,783 annotated healthy and pathological human tissue samples from publicly available Affymetrix microarray experiments. The database contains 15 healthy breast tissue samples and 1,504 different human breast carcinoma samples.

### Immunohistochemistry

Five different ready-to-use mouse or rabbit monoclonal antibodies were used from Ventana Medical Systems/Roche Diagnostics: Estrogen Receptor (SP1, rabbit), Progesterone Receptor (1E2, rabbit), HER-2/neu (4B5, rabbit), Ki-67 (30-9, rabbit), and p63 (4A4, mouse) with BenchMark XT immunostainer and *ultra*VIEW Universal DAB Detection Kit (Ventana/Roche, Tucson, Arizona, USA). The percentage of nuclei with immunoreactivity to ER, PR, and Ki-67 was classified as continuous data from 0 to 100 %. ER-positive and PR-positive cases showed staining in at least 10 % of the tumor cell nuclei. Carcinomas revealing 3+ immunohistochemical membrane staining for Her2 or positive gene amplification by CISH were accepted as positive (Boström et al. 2009). Immunostaining for p63 was done to identify myoepithelial cells in some cases. Immunohistochemical analyses for decorin were performed as previously described in detail (Salomäki et al. 2008).

### Decorin in situ hybridization

Decorin ISH was performed on 4 μm breast tissue sections by probing with human decorin antisense and sense single-stranded RNA riboprobes. A 533 bp fragment containing human decorin cDNA was cloned into the* Eco* RI/*Hin*d III site of pGEM-4Z transcription vector (kindly provided by Dr. Liliana Schaefer, University of Frankfurt, Frankfurt am Main, Germany). Linearized plasmid DNA was purified with QIAquick PCR Purification Kit (QIAGEN, Hilden, Germany) and DIG-labeled sense and antisense RNA probes were synthesized by in vitro transcription with SP6 and T7 polymerases, respectively, using a DIG RNA Labeling Kit (Roche, Applied Science, Mannheim, Germany). Probe quantification was carried out with a DIG Nucleic Acid Detection Kit (Roche), and ISH was performed as described (Salomäki et al. 2008).

### Adenoviral vectors

For transduction experiments, a recombinant replication-deficient adenoviral vector dcn-pxc1c-1 was used. This vector harbors the human decorin (*DCN*) cDNA under the control of cytomegalovirus (CMV) promoter. For the preparation of the vector, full length human decorin cDNA (Fisher et al. 1989) in pGEM plasmids was cloned and inserted into shuttle plasmid pxcJL-1. The viruses were prepared by cotransfecting HEK293–cells with back bone plasmid pBHG10. As a control vector RAdlacZ, which harbors the *E. coli* β-galactosidase gene (lacZ) under the control of CMV IE promoter was used (Wilkinson and Akrigg 1992). This vector was purchased from the Virus Vector Facility, Centre for Biotechnology, University of Turku, Turku, Finland.

### Decorin transduction

Human breast adenocarcinoma cell line MCF7 was used for transduction with a recombinant replication-deficient adenoviral vector dcn-pxc1c-1. MCF7 cells were maintained in RPMI-1640 medium containing 10 % fetal bovine serum (FBS), 25 μM insulin, 1 nM β-estradiol, 2 mM l-glutamine, penicillin (100 IU/mL), and streptomycin (100 μg/mL) and grown at 37 °C with 5 % CO_2_. The cells were plated on a 24-well plate (Greiner Bio-One, Kremsmuenster, Austria), 30,000 per well. The next day, cells were transduced with 0, 3, 30, 100, 300, and 1000 pfu/cell of dcn-pxc1c-1 or RAdlacZ in reduced medium containing no FBS. Four parallels were made of each vector concentration. After 24-h incubation, the cells were washed twice with reduced medium and incubated in this medium for another 24 h. The cells were trypsinized, pooled, and the RNA was extracted using NucleoSpin RNA II–kit (Macherey–Nagel, Düren, Germany) according to the manufacturer’s instructions.

### RT-qPCR

RNA concentration from the extractions was determined using a Nano-Drop spectrophotometer (ThermoScientific, Waltham, MA, USA), and the integrity of the RNA was confirmed with agarose gel electrophoresis. One μg of RNA was DNase treated with RQ1 RNase-Free DNase (Promega, Madison, WI, USA) and reverse transcribed into cDNA using M-MLV reverse transcriptase and Oligo(dT)15 primer (Promega, Madison, WI, USA) according to manufacturer’s instructions. RT-qPCR was performed using GoTaq qPCR Master Mix (Promega, Madison, WI, USA) with 100 nM primer concentrations and final volume of 10 μL according to manufacturer’s protocol. GNB2L1 was chosen as a reference gene (Zhang et al. 2005). Primer pairs used in qPCR were: 5′-GGACCGTTTCAACAGAGAGG-3′ (for) and 5′-GAGTTGTGTCAGG GGGAAGA-3′ (rev) for decorin and 5′-GAGTGTGGCCTTCTCCTCTG-3′ (for) and 5′-GCTTG CAGTTAGCCAGGTTC-3′ (rev) for GNB2L1. Reactions were run on an Applied Biosystems 7900HT machine (Applied Biosystems, Carlsbad, CA, USA). The qPCR protocol consisted of initial denaturation at 95 °C for 2 min followed by 40 cycles of denaturation at 95 °C for 40 s and extension at 60 °C for 45 s. The specificity of the reactions was confirmed by melt-curve and agarose gel analysis. Triplicate CT values were analyzed using the comparative CT (2^−△△CT^) method.

### Statistical analysis

Unpaired Student’s *t* test was employed in statistical analyses. All *p* values <0.05 were considered statistically significant.

## Results

### Relative decorin gene expression in human breast cancer tissues based on the GeneSapiens in silico transcriptomics data

**Fig. 1:**
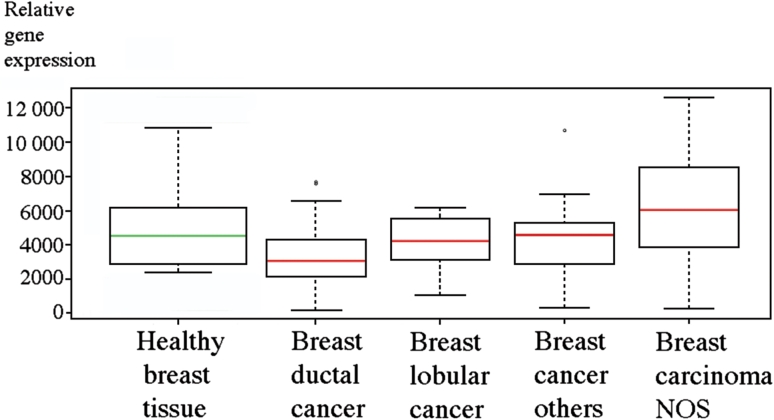
Box plot analysis of decorin gene expression levels using the GeneSapiens in silico database at www.genesapiens.org in healthy human breast tissues and in different types of human breast cancer. *Y* axis indicates the level of relative decorin gene expression in the tissues. The continuous lines in the box plot images represent the median gene expression level of decorin in breast tissue samples included in the database. Compared to decorin expression in healthy breast tissue decorin expression is decreased in ductal, lobular, and other breast cancers but increased in breast carcinomas not otherwise specified (breast carcinoma NOS)

In order to analyze published data on decorin gene expression in different types of human breast cancer, we used an in silico database from the GeneSapiens website (Kilpinen et al. 2008). The analysis indicated that the relative decorin gene expression is significant in both healthy and various malignant conditions of human breast tissue (Fig. 1).

### Localization of decorin mRNA in normal human breast tissue, and in benign and malignant tumors of the human breast

**Fig. 2:**
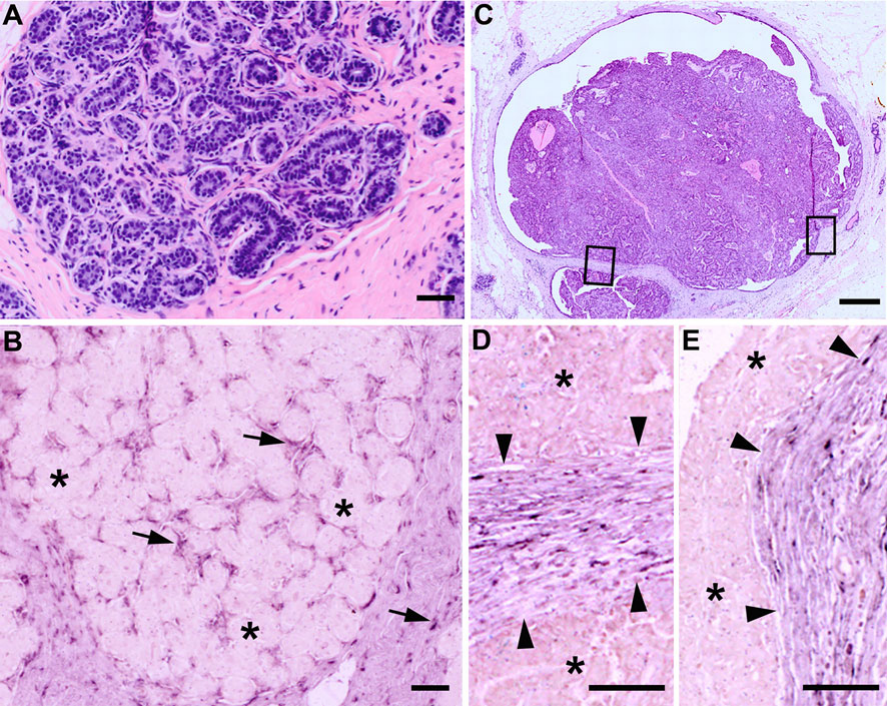
Decorin mRNA is localized solely to the stromal cells surrounding the lobulus and to the intralobular stromal cells of normal human breast tissue. **a** HE staining of normal lobulus and its stroma. **b** ISH for decorin of a serial section of the same normal lobulus as in (**a**). Examples of individual stromal cells expressing decorin mRNA are indicated by *arrows*. In the epithelium of ducts or lobules, no decorin mRNA can be detected (examples indicated by *asterisks*). **c** HE staining of human breast tissue containing intraductal papillomas. **d**, **e** ISH for decorin of a serial section of the same sample as in (**c**), magnified illustrations of the *boxed regions* shown in (**c**). Positive DIG-reaction in ISH can be seen in *purple*. No decorin mRNA is detected within the intraductal papilloma tissue (indicated by *asterisks*). *Arrowheads* indicate the borders between intraductal papillomas and normal breast tissue. **a**, **b** and **d**, **e** *scale bar* 50 μm, **c** *scale bar* 500 μm

**Fig. 3:**
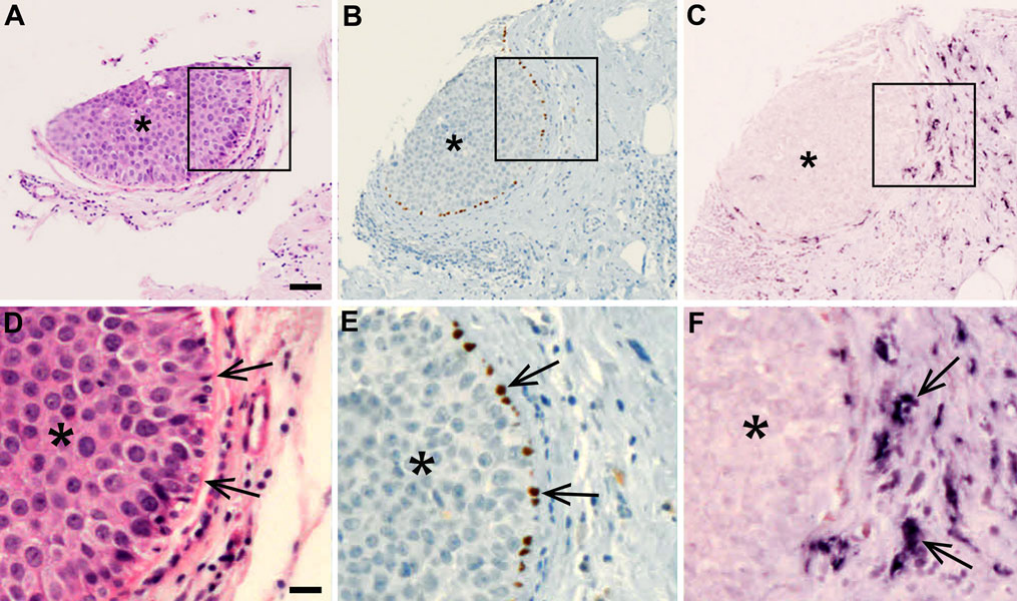
Decorin gene expression is lacking in human ductal carcinoma in situ tumor (DCIS). IHC and ISH of serial sections of a representative DCIS GII sample. Tumor tissue in the sections is indicated by an *asterisk*. **a**, **d** HE staining. **b**, **e** Immunostaining for p63. *Brown color* indicates p63 positive myoepithelial cells lining the tumor mass. **c**, **f** ISH for decorin. Positive DIG-reaction in ISH indicating the cells expressing decorin mRNA can be seen in *purple*. *Arrows* (**d**–**e**) indicate the border between DCIS and surrounding tissue. *Arrows* (**f**) indicate examples of decorin positive cells. **a**–**c** *scale bar* 100 μm, **d**–**f** *scale bar* 20 μm

**Fig. 4:**
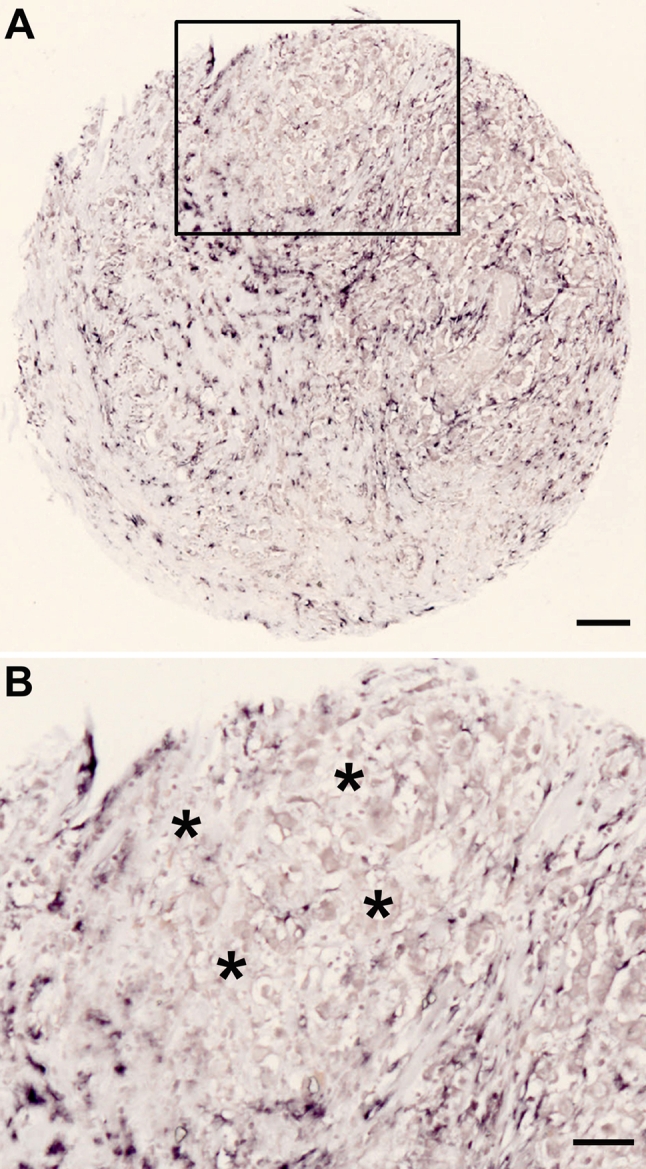
Cells of invasive ductal cancer (IDC) of human breast are negative for decorin mRNA. **a** ISH of an infiltrating ductal breast carcinoma GIII tissue sample. **b** Magnified illustration of the *boxed region* shown in (**a**). Positive DIG-reaction of stromal cells in ISH indicating the localization of decorin mRNA can be seen in *purple*. *Asterisks* in (**b**) indicate areas of infiltrating cancer cells that are negative for decorin gene expression. Note that all decorin mRNA within invasive cancer tissue sample is detected in the original stromal cells, not in the cancer cells. **a** *scale bar* 100 μm, **b** *scale bar *50 μm

**Fig. 5:**
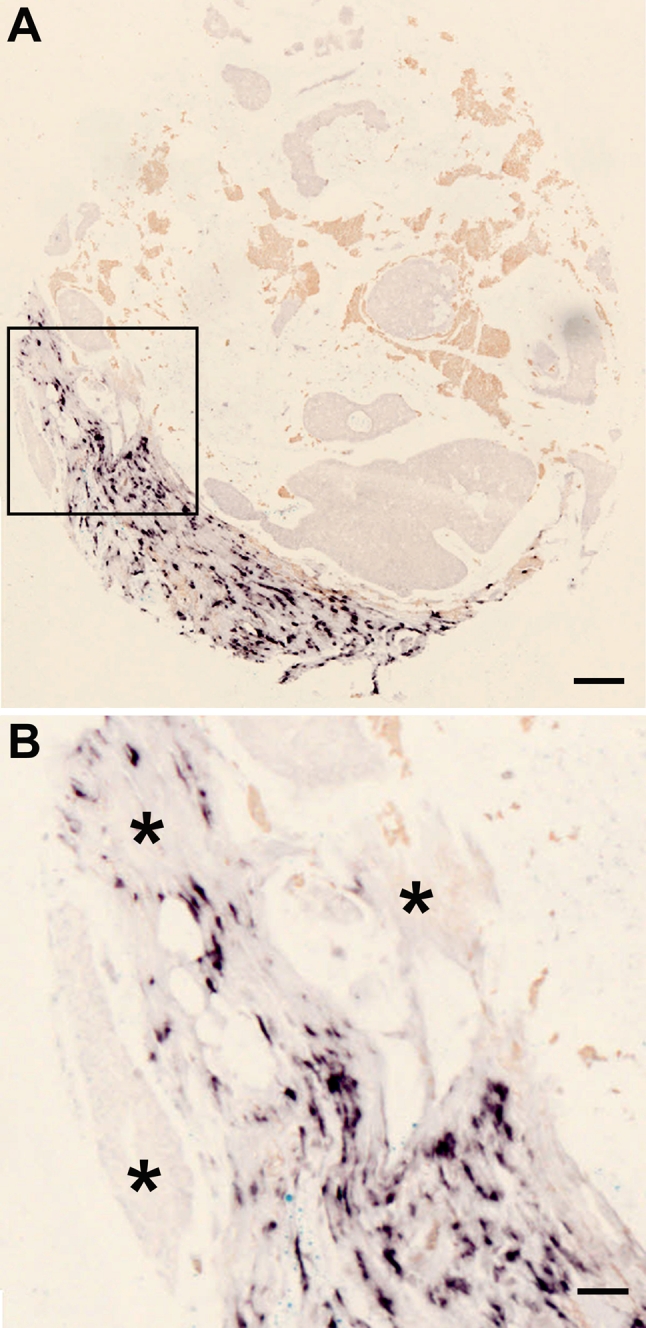
Invasive mucinous carcinoma cells of human breast lack decorin gene expression. **a** ISH for decorin of a representative invasive mucinous breast cancer tissue sample. **b** Magnified illustration of the *boxed region* shown in (**a**). Positive DIG-reaction in ISH indicating decorin expressing cells can be seen in *purple*. *Asterisks* in (**b**) indicate areas of invasive cancer cells. Note that these areas do not express decorin mRNA and all detected decorin mRNA is located in the original stromal cells. **a** *scale bar* 100 μm, **b** *scale bar* 50 μm

Next, we localized decorin mRNA in healthy human breast tissue, and in benign and selected types of malignant human breast tumors. Using ISH with DIG-labeled RNA probes for decorin, we were able to demonstrate that in healthy human breast tissue decorin gene expression takes place only in cells in the stromal area surrounding the lobules and in the intralobular stroma, whereas in cells of the epithelium of ducts or lobules no decorin gene expression was detected (Fig. 2a, b). Identical analysis of intraductal papillomas of the human breast (Fig. 2c) revealed that in these benign ductal tumors no decorin gene expression takes place (Fig. 2d, e). Similarly to the healthy human breast tissue, decorin mRNA was solely localized to the original stroma around the dilated duct of the papillomas and not at all within the papillomas (Fig. 2d, e). There was also no decorin mRNA detected in the area within breast tissue specimen containing malignant proliferation of ductal or lobular epithelial cells with myoepithelium, the so-called ductal carcinoma in situ (DCIS) (Fig. 3a–f), and lobular carcinoma in situ (LCIS) (data not shown). Indeed, in DCIS- and LCIS-containing samples, expression of decorin mRNA was localized merely to the peritumoral stroma. Decorin mRNA was also lacking from the infiltrating cancer cells of the invasive ductal carcinoma (IDC) (Fig. 4a, b) and the invasive lobular carcinoma (ILC) (data not shown). All decorin mRNA in the above-mentioned invasive human breast cancer samples was localized to the original stromal cells. Furthermore, in invasive mucinous carcinomas cancer cells in the mucin lakes or those within the original stroma did not express decorin mRNA (Fig. 5a, b). In conformity with IDC and ILC described above, the surrounding original stromal cells were highly positive for decorin mRNA. Positive immunoreactivity for decorin was seen in the same original non-malignant stromal area as decorin mRNA (data not shown).

### Effect of adenoviral decorin transduction on MCF7 cells

**Fig. 6:**
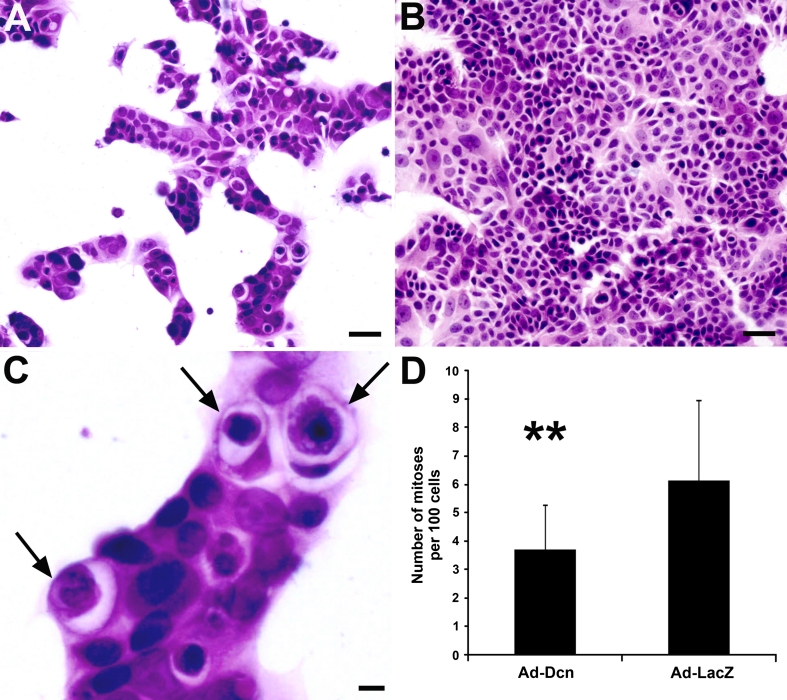
Decorin gene transduction mediated by a recombinant adenovirus has a significant effect on the growth pattern and proliferation of MCF7 cells. HE staining of MCF7 cell cultures transduced with human decorin cDNA-containing adenovirus (Ad-Dcn) (**a**) and lacZ gene-contaning adenovirus (Ad-LacZ) (**b**). **c** Magnified illustration of cannibal cells from figure (**a**). *Arrows* indicate the presence of cannibal cells after Ad-Dcn transduction of MCF7 cells. **d** Number of observed mitoses per 100 cells, 2 days after in vitro incubation with Ad vectors. **a**, **b** *scale bar* 50 μm, **c** *scale bar* 10 μm, **p < 0.001, Student’s *t* test

The ISH results clearly demonstrated that decorin is not expressed by benign or malignant ductal or lobular epithelial cells of the human breast. Therefore, next we wanted to examine the effects of targeted decorin transduction on the behavior of human breast adenocarcinoma cells. Human breast adenocarcinoma cell line MCF7 and decorin producing adenoviral vector were applied for this purpose. Using RT-qPCR, it was first shown that MCF7 cells do not express decorin mRNA (data not shown). These cells were then transduced with a titer range of 3–1,000 pfu/cell of a decorin expression vector and a viral concentration of 100 pfu/cell was chosen for further experiments. Transduction with a decorin adenoviral vector changed markedly the growth pattern of MCF7 cells. Cell cohesion clearly decreased and decorin-transduced MCF7 cells exhibited abnormal features (Fig. 6a), compared either to the cells transduced with a control vector LacZ (Fig. 6b) or to negative control cells (data not shown), which both grew as large cohesive solid sheets. Several of the decorin-transduced MCF7 cells were also found to contain a large vacuole within another larger cell with a crescent-shaped nucleus at its periphery (Fig. 6c). Microscopic examination further revealed apoptotic features in many of these internalized cells. These so-called cannibal cells were also sporadically seen among the MCF7 cells transduced with the control vector and among the MCF7 cells without any transduction (data not shown). The mitosis rate was statistically significantly lower in the decorin-transduced MCF7 cell cultures compared to their counterpart cell cultures treated with the control vector LacZ (Fig. 6d).

## Discussion

Previously, decreased level of decorin expression has been reported in breast cancer (Eshchenko et al. 2007; Gu et al. 2010; Leygue et al. 2000). However, in a recent study, even increased amounts of decorin in breast carcinoma has been observed (Skandalis et al. 2011). In addition to these conflicting results, it has also not been convincingly demonstrated whether breast cancer cells themselves express decorin or not (Cawthorn et al. 2012; Gu et al. 2010).

In the present study, we have first analyzed previously published data on decorin gene expression using GeneSapiens databank. Thereafter, we have localized decorin mRNA in tissues samples of normal human breast and selected human breast tumors using ISH with DIG-labeled decorin probes. We have also examined the influence of adenoviral mediated decorin transduction on the behavior of human breast cancer cells in vitro. GeneSapiens databank analysis demonstrated that the relative decorin gene expression is significant in both healthy and various malignant conditions of human breast tissue. ISH of healthy human breast tissue and selected human breast tumor samples revealed that decorin mRNA can merely be localized to the normal stroma. Indeed, no decorin mRNA could be detected in benign epithelial cells or in malignant cancer cells of the human breast. In previous studies, it has been demonstrated that decorin expression is high in normal human breast tissue stroma adjacent to lobules and reduced in the breast tumor itself (Brown et al. 1999; Leygue et al. 2000). However, these studies have used radioactive probes use of which is limited, if the purpose is to exactly localize the mRNA at the cellular level. With radioactive probes, all the cellular relationships are lost because of tissue digest and mRNA levels are averaged from all of the cells contained in the original sample (Wilcox 1993). When methods like Northern (Tralhão et al. 2003) or western blot analyses (Leygue et al. 2000; Nash et al. 2002; Troup et al. 2003) and more modern methods such as RT-PCR (Eshchenko et al. 2007) are used to evaluate the expression of molecules, e.g., decorin, they are usually performed with pooled samples consisting of the sectioned tumor which also contain its surrounding original stroma. Therefore, these above-mentioned methods including GeneSapiens database used in the present study are unable to tell the cellular origin of a specific molecule. Our results using ISH with DIG-labeled decorin probe have clearly shown that human breast cancer cells do not express decorin at all and that in human breast tissue specimen decorin is derived from original stromal cells, not from benign or malignant tumor-forming epithelial cells. However, the specific cell types responsible for decorin expression in the original stroma compartment still remains to be studied. To the best of our knowledge, this study is the first to exactly localize decorin mRNA at the cellular level in human breast tissue including different types of human breast cancer.

Next, we examined whether by introducing decorin, a known antitumoral molecule, to widely used MCF7 (Burdall et al. 2003) human breast adenocarcinoma cells, we could modulate the behavior of these cells. We showed that decorin transduction causes marked changes in the proliferation and growth pattern of the MCF7 cells. In particular, compared to control MCF7 cells, decorin-transduced MCF7 cells exhibited statistically significantly lower mitosis rate, and they also revealed increased apoptotic features such as the formation of the so-called cannibal cells. Earlier, it has been shown in mice that decorin gene delivery decreases the proliferative index (Pucci-Minafra et al. 2008) and alters the architecture and differentiation of the tumor xenografts, thereby retarding the growth of colon and squamous cell carcinomas (Reed et al. 2002). Using an animal model of orthotopic breast carcinoma, decorin has also been shown to slow mammary carcinoma cell motility, induce significant apoptosis and impede cell invasion through a three-dimensional extracellular matrix (Goldoni et al. 2008).

The mechanism(s) of action of decorin transduction on the behavior of MCF7 human breast cancer cells was not in our focus in this study and therefore remains to be explored. However, the observed decreased cell cohesion of MCF7 cells in response to decorin transduction could be caused by modulated pericellular matrix around the cells, because previously decorin has been shown to influence cellular adhesion via its capability to interact e.g., with fibronectin (Winnemöller et al. 1991) and thrombospondin (Winnemöller et al. 1992). It is also possible that alterations in the expression of metalloproteinases (MMPs) and their inhibitors (TIMPs) by MCF7 cells are responsible for the observed decreased cell cohesion. Indeed, adenovirus-mediated overexpression of decorin has been shown to modulate the expression of certain MMPs and TIMPs by human gingival fibroblasts (Al Haj Zen et al. 2003). Furthermore, the possibility that the decreased cell cohesion of MCF7 cells in response to decorin transduction is mediated via direct interactions between decorin and certain integrins remains to be explored (Guidetti et al. 2002). The decreased proliferation of decorin-transduced MCF7 cells, on the other hand, is most likely mediated via decorin’s ability to antagonize receptor tyrosine kinases, particularly the signaling through epidermal growth factor receptor (Hu et al. 2009), the receptor for hepatocyte growth factor called Met (Goldoni et al. 2009), insulin-like growth factor receptor (Iozzo et al. 2011) and/or vascular endothelial growth factor 2 receptor (Khan et al. 2011). In addition, induction of apoptotic features of MCF7 cells due to decorin transduction may also have a role in the finding of this study that decorin-transduced MCF7 human breast cancer cells exhibit decreased proliferation. Interestingly, exogenous decorin core protein has been shown to inhibit cancer growth by triggering apoptosis via activation of caspase-3 (Seidler et al. 2006). Finally, decorin’s ability to bind and block transforming growth factor-β should be kept in mind (Yamaguchi et al. 1990), although transforming growth factors have been shown not to act as major growth regulators of MCF7 cells (Herman and Katzenellenbogen 1994).

In conclusion, by utilizing ISH with DIG-labeled probes for decorin, we have shown that in human breast cancer, the cancer cells do not express decorin mRNA, but the expression of decorin takes place merely in cells of the original stroma both in healthy human breast tissue and in breast tissues containing benign or malignant breast epithelial tumors. By introducing a decorin producing adenoviral vector to the MCF7 human breast adenocarcinoma cells, we have also shown that decorin transduction in these decorin-negative cells results in marked changes in their behavior. Specifically, we have shown that decorin-transduced MCF7 cells exhibit decreased cell cohesion and statistically significantly lower mitosis rate and increased apoptotic features. Thus, our study provides evidence that targeted decorin transduction to breast cancer cells could be used as a novel adjuvant therapy in the treatment of human breast cancer in the future.
